# Perioperative Management of Patients with Cardiac Implantable Electronic Devices and Utility of Magnet Application

**DOI:** 10.3390/jcm11030691

**Published:** 2022-01-28

**Authors:** Tardu Özkartal, Andrea Demarchi, Maria Luce Caputo, Enrico Baldi, Giulio Conte, Angelo Auricchio

**Affiliations:** 1 Cardiocentro Ticino Institute, Ente Ospedaliero Cantonale, 6900 Lugano, Switzerland; andrea.demarchi@eoc.ch (A.D.); marialuce.caputo@eoc.ch (M.L.C.); enrico.baldi@unipv.it (E.B.); giulio.conte@eoc.ch (G.C.); angelo.auricchio@eoc.ch (A.A.); 2 Arrhythmia and Electrophysiology and Experimental Cardiology, Fondazione Istituto di Ricovero e Cura a Carattere Scientifico (IRCCS) Policlinico San Matteo, 27100 Pavia, Italy; 3 Faculty of Biomedical Sciences, Università della Svizzera Italiana (USI), 6900 Lugano, Switzerland

**Keywords:** cardiac implantable electronic device, perioperative management, magnet application, pacemaker, implantable cardioverter defibrillator, cardiac resynchronization therapy

## Abstract

With the demographic evolution of the population, patients undergoing surgery today are older and have an increasing number of sometimes complex comorbidities. Cardiac implantable electronic devices (CIED) are also getting more and more complex with very sophisticated programming algorithms. It may be generally assumed that magnet application reverts pacing to an asynchronous mode in pacemakers and disables tachycardia detection/therapy in internal cardioverter-defibrillators. However, depending on device type, manufacturer and model, the response to magnet application may differ substantially. For these reasons, perioperative management of CIED patients is getting more and more challenging. With this review article we provide an overview of optimal perioperative management of CIED patients with a detailed description of CIED response to magnet application depending on manufacturer and device-type, which may help in providing a safe perioperative management plan for the CIED patient.

## 1. Introduction

The clinical adoption of novel therapies which were developed over the last two decades, including cardiac implantable electronic devices (CIEDs) for patients with cardiovascular diseases, resulted in a substantially improved quality of life and survival of these patients. As a result, more CIED patients are possibly exposed to diseases in need of surgery during their lifetime [1,2,3]. Given the increasing complexity of CIED programming, peri- and intra-operative management of CIED patients by surgeons, anesthesiologists, internal specialists and cardiologists require a good understanding of the device function and behavior in case a CIED is exposed to electromagnetic interference (EMI) during surgery. Placing a magnet over the device may be sufficient for some devices, whereas for others it may cause unexpected CIED behavior or even harm the patient [4,5].

Anesthesiologists refer to clinical practice recommendations by the American Society of Anesthesiologists for the management of CIED patients [6]. These guidelines, however, do not specifically focus on CIED behavior and possible issues occurring during magnet application. On the other hand, cardiologists and clinical electrophysiologists refer to recommendations which were issued by the Heart Rhythm Society [7] more than 10 years ago. This document does not consider more recent device technologies such as subcutaneous implantable cardioverter defibrillators (ICD) or leadless cardiac pacemakers (LCP). Notably, to date, there are no European guidelines regarding the perioperative management of CIED patients. In contrast, national recommendations such as those issued jointly by the Austrian Societies of Anesthesiology, Cardiology and Surgery are published in their national language [8]; others, such as those for Switzerland [9], are published in national and not indexed journals.

In this review article, we provide an overview of perioperative management of CIED patients and summarize the CIED response to magnet application depending on device manufacturer, device type and its programming.

## 2. What Could Go Wrong?

The main issue for patients with CIEDs during surgery is EMI, which is most commonly caused by monopolar electrosurgery [10,11,12,13]. Bipolar electrosurgery [14], radiation therapy [15,16,17,18,19] and radiofrequency ablation [20,21,22] may also occasionally cause EMI-induced CIED malfunctions.

In pacing-dependent patients, EMI may lead to oversensing and inappropriate pacing inhibition with the risk of asystole [23,24]. In ICD patients, EMI may induce inappropriate anti-tachycardia pacing or defibrillation [25,26] which may cause sudden patient movement, possibly at a critical moment during surgery or may even induce a ventricular arrhythmia [27] with possible fatal outcome in extreme scenarios [28]. Very rarely, battery depletion has been reported as a result of repeated and unrecognized shock delivery during surgery [4]. Finally, there are several case reports of electrical reset to backup pacing mode after radiation therapy [15,16], radiofrequency ablation [29] and—in older devices—after the use of monopolar electrosurgery [7].

Further issues may arise when a rate response mode is activated in those devices which take advantage of minute ventilation for heart rate adaptation to exercise. Inappropriate rapid pacing [30,31] may occur as a consequence of altered impedance measurements due to current injection during electrosurgery or during mechanical ventilation [7]. A sudden increase in heart rate may have significant hemodynamic consequences, especially in patients with advanced heart failure. Therefore, meticulous preoperative assessment and surgical planning, possibly including CIED-reprogramming, are key for the device patients’ safety.

## 3. Device Type, Manufacturer, Model, Battery Longevity and Programming

**Figure 1:**
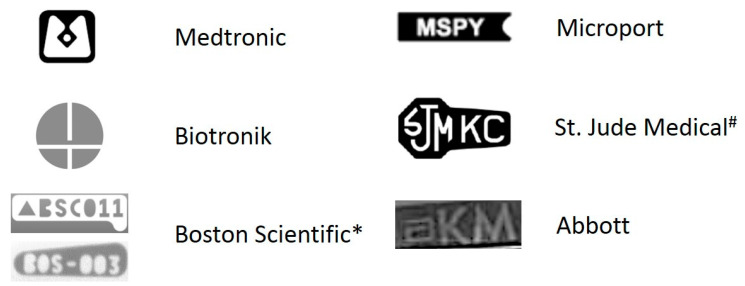
Radiopaque manufacturer identifier of the most frequently implanted CIEDs. Each manufacturer has a specific radiopaque identifier embedded within the device header which can be visualized by means of an X-ray of the chest. Depending on the model, MRI compatibility, etc., there may be some additional letters, numbers or symbols. # The vast majority of St. Jude Medical CIEDs show three specific letters, i.e., SJM, but additional letters may appear. * Boston Scientific CIEDs are labeled as either BSC or BOS, followed by an additional three-digit numeric code, which can vary according to the model.

Peri-operative management starts with the identification of the CIED indication, the type of implanted device and its programming. Most of this information is stated on the international identification card as well as on the device follow-up documents. In case this information is not available, a thoracic chest X-ray might help to identify the device manufacturer given the unique radiopaque identifier label which is localized on the device header (see Figure 1). Anyhow, if the identifier is not clearly visible, the CaRDIA-X algorithm which uses a stepwise approach by analyzing electrodes and generator shape, may be helpful [32]. Once the manufacturer is successfully identified, the device can safely be interrogated in order to obtain the information needed.

## 4. Pacemaker Dependency and Implantation Indication

Implantation indication may help to estimate the perioperative risk of significant bradycardia or asystole. For instance, patients with a high-grade AV block as indication to permanent pacing, have a significantly higher risk of being pacing dependent. Thus, the risk of developing asystole due to inappropriate pacing inhibition is higher compared to patients with sick-sinus syndrome or atrial fibrillation with a slow ventricular rate [33]. Therefore, it is advisable to assess pacing dependency, i.e., absence of an intrinsic rhythm at a programmed pacing rate of 30 ppm [33], when indication to pacing is unclear, unknown or when the last device follow-up is older than 6 months. In patients who underwent ICD implantation following a history of ventricular tachycardia or ventricular fibrillation (the so-called secondary prevention of sudden cardiac death indication), the risk of developing perioperative malignant arrhythmias may be higher compared to patients with a primary prevention ICD indication. 

## 5. Risk of EMI and Measures to Minimize Its Occurrence

As a general rule, one may state that the further the operation site and the dispersive electrode is away from the CIED, the less the EMI risk. In a recently published study, EMI occurrence during surgery within 15 cm from the CIED generator amounted to 47%, whilst it was only 7% for abdominal/pelvic and 0% for lower abdominal or inferior extremity procedures [34]. These findings are consistent with other studies, confirming low EMI risk in surgeries below the iliac crest [10,11,13,35]. However, there is anecdotal evidence of inappropriate shock delivery despite a distant surgery site [36] because of inconvenient placement of the electrosurgery dispersive electrode. A recent study analyzed a standardized approach of dispersive electrode placement aiming to maximize the distance between current flow from electrosurgery to dispersive electrode and CIED generator/electrodes [13]. EMI occurrence causing inappropriate therapies amounted to 29% for cardiac surgery, 7% for non-cardiac surgery above and 0% below the umbilicus. The authors found that EMI occurrence would have been further reduced to 8.8% for cardiac surgery and to 2.9% for non-cardiac surgery above the umbilicus by systematically programming ICDs according to current recommendations, i.e., high detection rates and/or long detection duration [37,38]. Correct dispersive electrode positioning and ICD programming is therefore crucial do reduce EMI risk [39]. EMI is less frequent in bipolar electrosurgery and, whenever possible, it should be preferred over monopolar electrosurgery [9]. Since VT detection and therapy delivery by the ICD takes more than 5–10 s, electrical bursts should ideally be short (<5 s) with sufficient pauses (>5 s) in-between applications [9,40] so as to minimize occurrence of inappropriate therapy.

Finally, one should be aware of the fact that EMI risk is higher in ICDs than in PM because the ventricular channel is programmed in a very sensitive manner in order to assure adequate recognition of ventricular fibrillation with low amplitudes. Risk of EMI is also higher in integrated bipolar leads, as opposed to true bipolar leads, due to the larger inter-electrode spacing between the tip electrode and the coil (larger antenna) [13].

It is noteworthy that the manufacturers have developed so-called noise reversion algorithms aiming to reduce CIED malfunction due to electrical artefacts (noise), such as those caused by EMI. However, despite these algorithms, EMI induced malfunction continues to be an important issue.

## 6. Pacemaker and Cardiac Resynchronization Therapy—Pacemakers (CRT-P)

As a general rule, when a magnet is applied, PM and CRT-P devices convert their pacing to an asynchronous mode (i.e., VOO for single chamber, and DOO for dual-chamber devices) at magnet rate. The magnet rate depends on battery status and differs between manufacturers (see Table 1 and Table 2). Pacing polarity (bi- or unipolar) remains unchanged; the sensor or “R function” is disabled. In case the device has automatically switched to a non-tracking mode, i.e., to VVIR or VDIR, due to ongoing atrial arrhythmia (so-called automatic mode switch) and a magnet is applied, the pacing converts to DOO. Hence, atrial spikes may be recorded on the surface ECG, even though the patient is in atrial fibrillation. This should not be mistaken for a CIED dysfunction. Although general response to magnet application is relatively similar among PM and CRT-P produced by different manufacturers, there are several device-specific behaviors one should be familiar with.

### 6.1. Abbott

Magnet response is programmable. The nominal setting is *Battery Test* and the magnet converts the device to asynchronous pacing, which may take up to five seconds, since an EGM is registered beforehand. If *AutoCapture* is enabled, the device goes to high-output mode. AV delay is set to 120 ms and in CRT-P, the delay between left and right ventricular pacing (VV-delay) remains unchanged. The magnet response can be programmed *Off*, in which case the magnet has no effect on the device function.

### 6.2. Biotronik

In case the magnet response is programmed to *asynchronous*, the pacing mode is converted to asynchronous pacing throughout magnet application with an AV delay of 100 ms. In case the magnet response is programmed to *synchronous*, pacing remains unaltered if the battery is sufficient; in case of ERI, pacing converts from DDD(R) to VDD and pacing rate decreases by 11%; in case DDI(R) or DVI(R) is programmed chronically, VA interval is extended by 11%, which reduces pacing rate by 4.5 to 11% (depending on programmed AV delay). In case *patient trigger* is programmed (nominally deactivated), magnet function is automatically set to *synchronous* and magnet application only triggers an EGM recording. Nominally, however, magnet response is *automatic*, which causes asynchronous pacing for only 10 cycles. Afterwards pacing converts automatically back to the original programming.

Therefore, for Biotronik pacemakers and CRT-P, magnet response should be evaluated prior to surgery by applying a magnet for 30 s in order to confirm continuous asynchronous pacing throughout magnet application. In case pacing converts back to a synchronous mode after 10 cycles, it is preferable to reprogram the device to an *asynchronous* magnet response rather than to an asynchronous mode per se, as this eliminates the need for post-operative device reprogramming. Furthermore, in CRT-Ps, magnet application leads to right ventricular pacing only, causing loss of cardiac resynchronization. This might have negative hemodynamic effects.

### 6.3. Boston Scientific

Magnet response is nominally programmed to *Pace Async* (or *Async* in older models) and magnet application converts pacing to asynchronous mode with an AV delay of 100 ms. In CRT-Ps pacing is set to simultaneous biventricular pacing (LV Offset 0 ms), which may lead to asynchronous ventricular contraction due to intraventricular conduction delay and/or latency [41]. This may have negative hemodynamic consequences, especially in long-lasting surgeries. After magnet application, the pulse width of the third impulse is reduced by 50% to check for sufficient pacing safety margin; in case capture is lost, device interrogation is advisable.

In case magnet response is programmed to *Store EGM* (or *EGM*) magnet application leads to EGM storage without any effect on pacing. However, only one EGM is stored and afterwards (or after 60 days without magnet application), magnet response converts automatically to *Pace Async*. Finally, magnet response may be programmed *Off*.

### 6.4. Medtronic

In past generation pacemakers (Adapta, Versa, Sensia, Relia, Attesta), magnet application initiates a *threshold margin test:* pacing at 100 ppm with an AV delay of 100 ms and reduction in pacing amplitude by 20% on the third impulse in all paced chambers, followed by pacing at magnet rate. In case the third impulse does not capture the myocardium, device interrogation is advisable. In other models (Advisa, Ensura, Consulta, Syncra, Viva CRT-P) magnet application converts pacing mode to asynchronous pacing at magnet rate and AV interval is set to either the permanent programmed paced AV delay or 180 ms (whichever is shorter). In the most recent pacemakers (Azure, Astra) and CRT-P (Percepta, Serena, Solara), magnet application initiates asynchronous pacing at 100 ppm for 5 beats, followed by pacing at magnet rate. Note that in all CRT-Ps the magnet does not alter VV-interval. 

### 6.5. Microport

Magnet application changes the pacing mode to asynchronous mode, and increases pacing amplitude to 5 V with a pulse width of 0.5 ms in each paced chamber, including the left ventricle in CRT-P. An increase in pacing amplitude at the left ventricular site may become an issue causing diaphragmatic stimulation. On exiting magnet mode, the device paces six cycles at magnet rate in asynchronous mode with an AV delay of 95 ms, followed by two asynchronous cycles with permanently programmed parameters, before returning to permanent programming. During magnet application, VV delay is automatically set to 0 ms and AV delay to rest AV, which may, as mentioned before, cause ventricular asynchrony.

## 7. Implantable Cardioverter Defibrillator (ICD) and Cardiac Resynchronization Therapy—Defibrillator (CRT-D)

Magnet application deactivates tachycardia detection and/or anti-tachycardia therapy without influencing bradycardia pacing. Although in most ICDs and CRT-Ds, the pacing mode, sensor function, pacing polarity and intervals remain unchanged, the following section—as well as Table 3 and Table 4—show device-specific programming features in detail.

### 7.1. Abbott

Magnet response is nominally set to *Normal* and the magnet response is as expected. It may, however, be programmed to *Ignore*, in which case the magnet has no effect on the device. In newer models (Avant, Gallant, Entrant, Neutrino), magnet application causes the emission of an auditory tone for 4 s, thus confirming correct magnet positioning and deactivation of tachycardia detection and therapy. Magnet removal causes a higher audible tone for 6 s which confirms the termination of magnet mode. Both tachycardia detection and therapy delivery resume upon completion of the tone. This delay may become relevant in case a ventricular arrhythmia occurs during surgery.

### 7.2. Biotronik

The only noteworthy, peculiar feature is that after eight hours of continuous magnet application, tachycardia detection and therapy is automatically re-enabled. This should be considered in case of longer surgeries or in case a magnet is applied during postoperative or intensive care unit surveillance to disable tachycardia detection/therapy, for instance if a cardiologist is not readily available to reprogram the device. To avoid automatic re-activation, the magnet needs to be removed for a couple of seconds and repositioned before the eight-hour period expires.

### 7.3. Boston Scientific

At nominal setting, magnet response is programmed to *Inhibit Therapy* and the device enters a “monitor only” mode when a magnet is applied, i.e., detection of arrhythmias remains active, but therapies are disabled. Magnet response can be programmed *Off* or to *Store EGM* (note that after an EGM storage or 60 days without magnet application, magnet response automatically converts to *Inhibit Therapy*). If the device is permanently programmed to a non-therapy mode (*Off, Monitor Only* or *Electrocautery Protection*), magnet application causes a continuous tone. If it is programmed to *Monitor + Therapy* an R-synchronous beep (Confient, Vitality, Livian), or one beep per second (remaining models) will be emitted for as long as the magnet is placed over the CIED generator. Therefore, correct positioning may be confirmed acoustically throughout the entire time of magnet application, minimizing the risk of unnoticed magnet dislocation during surgery. Finally, *Electrocautery Protection* mode can be programmed, which converts bradycardia pacing to an asynchronous mode and temporarily deactivates tachycardia therapy. If a magnet is applied on a device programmed in *Electrocautery Protection* mode, a beeping tone will be emitted to indicate the absence of tachycardia therapy.

### 7.4. Medtronic

If *Patient Alerts* are not intentionally deactivated, an acoustic signal is emitted when a magnet is placed over the device. Continuous signal for 10 s means normal device function, intermittent on–off tone for 30 s (like a “truck backing up”) corresponds to a low urgency alert and alternating high–low frequency tone for 30 s (like a “French police car”) indicates presence of a high-urgency alert. Note that clinician-defined urgencies may be programmed as low- or high-urgency alerts and may be turned off, whereas system-defined alerts, such as ERI, are always high-urgency. Because of the emitted tone, correct positioning of a magnet over the device and hence deactivation of tachycardia detection and therapy is confirmed. However, as opposed to other devices, the duration of the acoustic signal is limited and magnet dislocation during surgery may still occur without being noticed by the surgical team.

### 7.5. Microport

Microport high-voltage devices are the only ones in which magnet application alters pacing, by increasing pacing amplitude to 6 V and pulse width to 1 ms in each paced chamber. As in CRT-P, this may become an issue, possibly causing diaphragmatic capture. Pacing rate is not altered by the magnet, except in older models (Paradym and Intensia), where it is set to magnet rate. Furthermore, as opposed to all other manufacturers, magnet application disables the sensor; hence, sensor-induced inappropriate rapid pacing cannot occur. Magnet removal initiates exiting mode as described in the PM/CRT-P section.

Another device feature which is unique to Microport high voltage devices is that therapy inhibition may be extended up to 2.5 min after magnet removal when a charge occurred just before magnet application. This should be considered when magnet is removed.

## 8. Leadless Pacemakers

To date, there are only two market-released leadless cardiac pacemakers: the Micra by Medtronic, and the Nanostim by St. Jude Medical. The functionality of the Medtronic leadless pacemaker is not affected by magnet application and the device must therefore be reprogrammed in pacing-dependent patients. The Nanostim leadless pacemaker by St. Jude Medical changes to an asynchronous mode (VOO), unless magnet mode is programmed *Off*; after eight cycles of pacing at 100 ppm, the device stimulates at magnet rate (90 ppm before recommended replacement time and 65 ppm thereafter). Note that pulse duration and amplitude are not altered, and no audible tone is emitted. After magnet removal the device reverts to previous programming within five seconds. It should be noted that the Nanostim device was recalled in 2016 owing to rare but serious battery failures and is rarely encountered in daily clinical practice. The new model, Aveir, which has not been released on the market yet, also converts to VOO mode at battery rate.

## 9. Subcutaneous ICDs

The only market released subcutaneous ICDs are from Boston Scientific. As in other ICDs, magnet placement over the device deactivates tachycardia therapy for as long as the magnet is applied. In first-generation S-ICDs (SQ-RX), the magnet needs to be positioned centrally over the device, whereas in the newer generations (Emblem and Emblem MRI), the magnet needs to be placed over the device header or the lower edge. The device emits an R-wave synchronous beep for 60 s to confirm magnet mode. If no audible feedback is present during magnet placement, this may indicate that magnet is not positioned correctly over the device, thus all therapies are still active; magnet repositioning usually resolves the issue. Another reason for absence of an audible tone at the time of magnet application is intentional deactivation during a previous follow-up visit. Finally, the lack of audible tone during magnet placement may be a malfunction of the tone emitter, which may be damaged by a strong magnetic field such as magnetic resonance imaging. Therefore, if beeping tone is absent despite correct magnet placement, it may be advisable to interrogate the device.

## 10. Perioperative CIED Management Strategies

**Figure 2:**
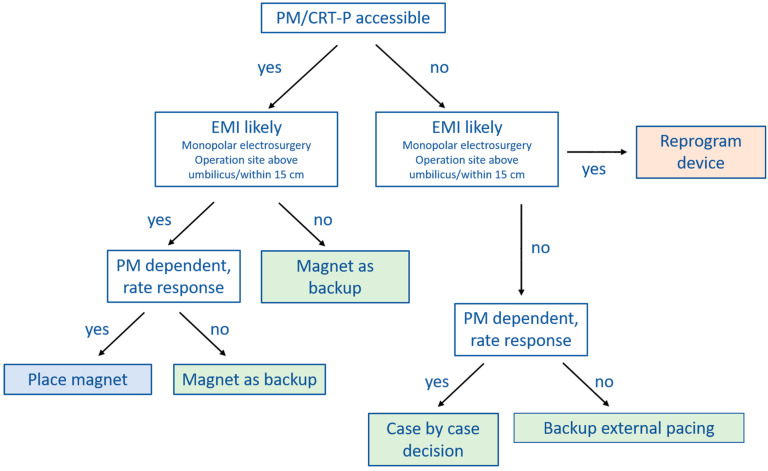
Proposed algorithm for perioperative management of patients with PM/CRT-P. First, check if generator is accessible and correct magnet positioning may be guaranteed throughout surgery (PM accessible). Afterwards check if EMI occurrence is likely. Finally, check if patient is pacing dependent. In case of preoperative programming, continuous rhythm monitoring is mandatory until device is reprogrammed. Correct placement of the dispersive electrode is crucial to reduce EMI risk. Consider specific magnet responses of different manufacturers and CIED models. In case invasive blood pressure monitoring is not available, pulsoxymetry should be used, since evaluation of monitor ECG may be difficult due to artefacts caused by electrosurgery. Reprogram device: program asynchronous mode; EMI—electromagnetic interference; PM—pacemaker; PM dependent—pacing dependent; CRT-P—cardiac resynchronization therapy—pacemaker.

**Figure 3:**
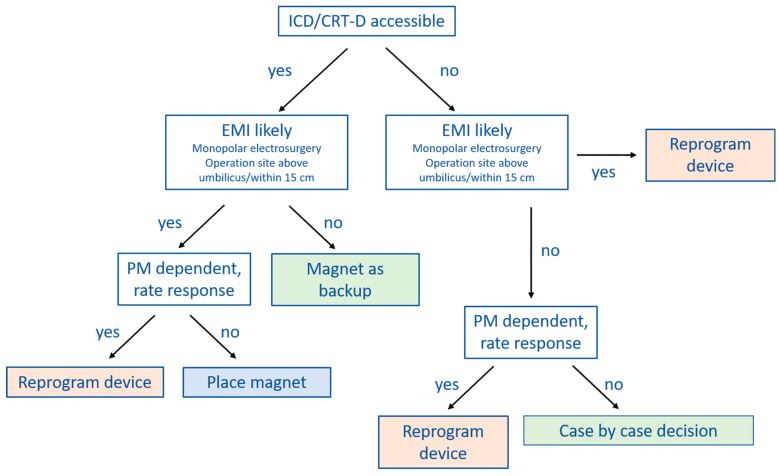
Proposed algorithm for perioperative management of patients with ICD/CRT-D. First, check if generator is accessible and correct magnet positioning may be guaranteed throughout surgery (PM accessible). Afterwards check if EMI occurrence is likely. Finally, check if patient is pacing dependent and if rate response is programmed. In case of preoperative programming, continuous rhythm monitoring is mandatory until device is reprogrammed. Correct placement of the dispersive electrode is crucial to reduce EMI risk. Consider specific magnet responses of different manufacturers and CIED models. In case invasive blood pressure monitoring is not available, pulsoxymetry should be used, since evaluation of monitor ECG may be difficult due to artefacts caused by electrosurgery. Reprogram device: deactivate tachycardia detection and therapy if patient is not pacing dependent; program asynchronous mode if patient is pacing dependent (automatically deactivates tachycardia detection and therapy); deactivate rate response, if necessary. EMI—electromagnetic interference; PM—pacemaker; PM dependent—pacing dependent; ICD—internal cardioverter defibrillator; CRT-D—cardiac resynchronization therapy—defibrillator.

Several clinical practice guidelines clearly recommend magnet application over CIED programming at the time of surgery [9,40], whereas others are less clear [6,7]. In Figure 2 and Figure 3 we provide practical flowcharts which might help with the selection of management strategy for CIED patients. Peri-operative management strategies of CIEDs may be summarized as follows: 

(1) No change in device programming, no magnet application: most of the currently available guidelines do not explicitly recommend this approach [6,7,9,40]. However, in case the surgery is below the iliac crest, or no electrosurgery or radiofrequency energy is used, close monitoring with defibrillator patches in place and a magnet readily available in the operation theater may suffice.

(2) Device programming prior to surgery rather than magnet application: there are a number of situations during which CIED programming should be preferred over magnet application. Since the magnet has no effect on pacing in most ICDs and CRT-Ds, device programming should be preferred in pacing-dependent patients or when the sensor (R-function) is activated. Furthermore, if the CIED is not accessible for magnet placement because the operation site is near the CIED generator or there is risk of potential shifting of the magnet due to the patient position (for example left lateral or prone position), reprogramming is also the preferred choice. 

However, there are also several issues associated with perioperative CIED programming that should be considered. The programming is usually performed by a cardiologist, who may not always be readily available thus causing delays and interrupting perioperative workflow. Furthermore, pre-operative CIED programming bears a certain risk of accidental post-operative non-reprogramming. Finally, once tachycardia detection and therapy are deactivated or the device is programmed to a (potentially pro-arrhythmic) asynchronous pacing mode [42,43], the patient needs to be continuously monitored. This may render perioperative coordination and management more complex. 

(3) Prophylactic magnet application: the main advantage of magnets is that they can easily be removed. In case of ventricular arrhythmias during surgery of an ICD patient, for instance, magnet removal reactivates tachycardia detection and therapy, avoiding the need for external defibrillation. Since ATP is effective in converting VT in 80–90% [41,44,45], defibrillation may even be avoided altogether. Other than being pro-arrhythmic, an asynchronous pacing mode may also cause competition between pacing and the patient’s intrinsic rhythm. This might cause hemodynamic repercussions, especially in heart-failure patients. Magnet removal immediately resolves this issue. Generally, seen the scenarios mentioned above, magnet application is preferred over device reprogramming whenever possible.

However, magnet application may also be problematic since it might slip during surgery without being noticed [36]. Furthermore, as discussed extensively before, magnet response may differ substantially among manufacturers and models. It is therefore crucial that the surgeon, anesthesiologist and cardiologist are aware of these differences.

## 11. Postoperative Device Management

Once the magnet is removed, the initially programmed setting is usually restored. In case the device is pre-operatively programmed to asynchronous pacing or tachycardia detection and therapy is disabled, it is mandatory to continuously monitor the patient until device reprogramming [6,9]. In case of suspected ICD therapy (ATP or defibrillation) or major events during surgery, such as cardiac arrest or external cardioversion/defibrillation, emergency surgery with EMI exposure above the umbilicus or cardiac surgery, or if there is suspicion of an electrical reset (see Table 5) [6,7] device interrogation should be performed before the patient leaves the monitored environment. Otherwise, routine follow-up is sufficient.

## 12. Conclusions

Perioperative management of patients with CIEDs can be challenging because EMI may cause device malfunction. To avoid CIED-related peri- and intra-operative complications, it is of the utmost importance to assess indication to device implantation, evaluate current pacing dependency, check the device’s last follow-up chart, and possibly contact patients’ electrophysiologist or their device specialist. All this information will help implement the most convenient strategy for a safe surgical approach.

## Figures and Tables

**Table 1:** Magnet application on Pacemakers.

Manufacturer	Magnet Mode, Rate at BOL/ERI (ppm)	Magnet Response Programmable	AV-Delay (if DDD)	Remarks
Abbott (former SJMl)	DOO/SOO, 100 ^§^/85 ^$^	*Battery test*^n^, *Off*	120 ms	Asynchronous pacing starts after EGM storage (may take up to 5 s) *AutoCapture* enabled: high-output mode
Biotronik	DOO/SOO, 90/80 *	*Auto* ^n^ *, Asynchronous, Synchronous*	100 ms	*Auto*: asynchronous pacing only for 10 cycles *Trigger function* enabled: magnet mode automatically set to *synchronous* Device in mode switch: DOO (only for 10 cycles if magnet mode is *Auto* ^n^) Dual-chamber pacemaker programmed to VVI: VOO (only for 10 cycles if magnet mode is *Auto* ^n^)
Boston Scientific	DOO/SOO, 100 ^%^/85	*Pace Async*^n^, *Store EGM, Off* (In older models: *Async*^n^, *EGM* or *Off*)	100 ms	*Pace Async*: pulse width of 3rd impulse reduced by 50% to evaluate sufficient pacing safety margin
Medtronic	DOO/SOO, 85/65	No	minimum pro-grammed pAV delay or 180 ms	Azure, Astra: 100 ppm for 5 cycles followed by magnet rate Adapta, Versa, Sensia, Relia, Attesta: *threshold margin test:* 100 ppm with 100 ms AV delay and amplitude reduction by 20% at 3^rd^ impulse, afterwards conversion to magnet rate Leadless cardiac pacemaker (MICRA VR and MICRA AV): magnet has no effect
Microport (former Sorin)	DOO/SOO, 96/80	No	resting AV delay	Pacing with 5 V @ 0.5 ms in each paced chamber (if not programmed higher) Exiting magnet mode: 6 cycles at magnet rate with 95 ms AV delay, followed by 2 asynchronous cycles with permanently programmed parameters, followed by permanent programming

**^§^** Exceptions: Affinity, Integrity, Identity, ADx, Victory, Zephyr: magnet rate 98.6 ppm at BOL and 86.3 ppm at ERI. Trilogy, Synchrony, Solus, Paragon, Phoenix: asynchronous pacing at permanently programmed rate. Leadless PM (Nanostim): 90 ppm at BOL, 65 ppm at RRT. ^$^ Gradual decrease from 100 ppm at BOL, via 85 ppm at ERI to 60 ppm at EOL. ^n^ Nominal programming. * *Synchronous*: at ERI: DDD→VDD, programmed heart rate decreased by 11%; DDI(R) or DVI(R)→VA interval extended by 11% (= pacing rate reduces by 4.5–11%, depending on programmed AV delay). **^%^** 100 ppm: battery duration > 1 year (in older devices = *GOOD*); 90 ppm: battery duration ≤ 1 year (in older devices = *ERN*); 85 ppm: generator replacement indicated (in older devices *ERT*). AV—atrioventricular, BOL—beginning of life, DOO—asynchronous dual-chamber pacing, EGM—intracardiac electrogram, EOL—end of life, ERI—elective replacement indicator, ERN—elective replacement near, ERT—elective replacement time, pAV—paced atrioventricular, ppm = pace per minute, RRT—recommended replacement time, SJM—St. Jude Medical, SOO—asynchronous single chamber pacing (either AOO or VOO).

**Table 2:** Magnet application on cardiac resynchronization therapy—pacemakers.

Manufacturer	Magnet Mode, Rate at BOL/ ERI (ppm)	Magnet Response Pro-grammable	AV-Delay (If DDD)	VV-Delay	Remarks
Abbott (former SJM)	DOO/VOO,100 ^§^/85 ^$^	*Battery test*^n^, *Off*	120 ms	not altered	Asynchronous pacing starts after EGM storage (may take up to 5 s)
Biotronik	DOO/VOO,90/80 *	*Auto*^n^*, Asynchronous*, *Synchronous*	100 ms	RV pacing only	*Trigger function* enabled: pacing mode automatically set to synchronous pacing
Boston Scientific	DOO/VOO,100 ^%^/85	*Pace Async*^n^, *Store EGM,Off* (In older models: *Async*^n^, *EGM, Off*)	100 ms	0 ms	*Pace Async*: pulse width of 3rd impulse reduced by 50% to evaluate sufficient pacing safety margin
Medtronic	DOO/VOO,85/65	No	Minimum of programmed paced AV delay or 180 ms	not altered	Percepta, Serena, Solara: pacing at 100 ppm for 5 beats followed by magnet rate
Microport (former Sorin)	DOO/VOO,96/80	No	rest AV delay	0 ms	Pacing output 5 V @ 0.5 ms (if not programmed higher) in each paced chamber Exiting magnet mode: 6 cycles at magnet rate with 95 ms AV delay and programmed output, followed by 2 asynchronous cycles with permanently programmed parameters, followed by permanent programming

**^§^** Exceptions: Affinity, Integrity, Identity, ADx, Victory, Zephyr: magnet rate 98.6 ppm at BOL and 86.3 ppm at ERI. Trilogy, Synchrony, Solus, Paragon, Phoenix: asynchronous pacing at permanently programmed rate. Leadless PM (Nanostim): 90 ppm at BOL, 65 ppm at RRT. ^$^ Gradual decrease from 100 ppm at BOL, via 85 ppm at ERI to 60 ppm at EOL. ^n^ Nominal programming. * *Synchronous*: at ERI: DDD→VDD, programmed heart rate decreased by 11%; DDI(R) or DVI(R)→VA interval extended by 11% (= pacing rate reduces by 4.5–11%, depending on programmed AV delay). **^%^** 100 ppm: battery duration > 1 year (in older devices = *GOOD*); 90 ppm: battery duration ≤ 1 year (in older devices = *ERN*); 85 bpm: generator replacement indicated (in older devices *ERT*). AV—atrioventricular, BOL—Beginning Of Life, DOO—asynchronous dual-chamber pacing, EGM—intracardiac electrogram, ERI—elective replacement Indicator, ERN—elective replacement near, ERT—elective replacement time, ppm = pace per minute, RRT—recommended replacement time, SJM—St. Jude Medical, VOO—asynchronous ventricular pacing. VV-Delay: interventricular pacing delay.

**Table 3:** Magnet application on an implantable cardioverter defibrillator.

Manufacturer	Tachycardia Function	Brady Function and Sensor	Magnet Response	Acoustic Signal	Remarks
Abbott (former SJM)	Detection and therapy inhibited	Not altered	*Normal*^n^, *Ignore*	Magnet mode initiation: 4 s tone * Magnet mode termination: 6 s higher tone *	Acoustic signal only in newer models (Avant, Gallant, Entrant, Neutrino)
Biotronik	Detection and therapy inhibited	Not altered	Not programmable	None	8 h of continuous magnet application: tachy detection and therapy automatically re-enabled
Boston Scientific	Therapy inhibited, detection active	Not altered	*Inhibit* *Therapy*^n^, *Off, Store EGM*	*Off, Monitor Only, Electrocautery Mode*: constant tone *Monitor + Therapy*: beeping tone S-ICD: 60 s beeping confirms deactivation of tachy detection and therapy PRIZM, PRIZM 2, VITALITY change from beep to continuous—therapies deactivated; change from continuous to beep—therapies re-activated;	*Inhibit Therapy*: detection remains active *Store EGM*: after 60 d or EGM storage via magnet: conversion to *Inhibit Therapy* Correct magnet positioning for S-ICD: centrally in SQ-RX 1010, over header or lower edge in Emblem. Older models (PRIZM, PRIZM 2, VITALITY): magnet toggles mode between *Monitor + Therapy* and *Off*; magnet repositioning required to change between modes
Medtronic	Detection and therapy inhibited	Not altered	Not programmable	10 s continuous: normal function 30 s intermittent on-off (“truck backing up”): low-urgency alert 30 s alternating high-low frequency (“French police car”): high-urgency alert	
Microport (former Sorin)	Detection and therapy inhibited	Altered (see remarks)	Not programmable	None	Pacing with 6 V @ 1 ms for each chamber Paradym, Intensia: pacing at magnet rate; exiting magnet mode: 6 cycles at magnet rate and 95 ms AV delay→2 asynchronous cycles as permanently programmed→permanent programming Sensor (R-function) is disabled Therapy Inhibition may extend up to 2.5 min after magnet removal if a charge occurred just before magnet application Device in mode switch: pacing as permanently programmed regardless of underlying rhythm *SafeR*: pacing at DDD regardless of AV conduction

* Avant, Gallant, Entrant, Neutrino. ^n^ Nominal programming. AV—atrioventricular; BOL—Beginning OF Life, d –day(s); EGM—intracardiac electrogram; EOL—End Of Life; h = hour(s); s—second(s); S-ICD—subcutaneous implantable cardioverter defibrillator; SJM—St. Jude Medical; Tachy—tachycardia.

**Table 4:** Magnet application on cardiac resynchronization therapy—defibrillators.

Manufacturer	Magnet Mode	Bradycardia Function (Including Sensor)	Magnet Response	AV/VV Delay	Acoustic Signal	Remarks
Abbott (former SJM)	Detection and therapy inhibited	Not altered	*Normal*^n^, *Ignore*	Not altered	initiation: 4 s tone * termination: 6 s higher tone *	Acoustic signal only in newer models (Avant, Gallant, Entrant, Neutrino)
Biotronik	Detection and therapy inhibited	Not altered	Not programmable	Not altered	None	8 h of continuous magnet application: tachy-detection/the-rapy automatically re-enabled
Boston Scientific	Therapy inhibited, detection active	Not altered	*Inhibit Therapy*^n^, *Off, Store EGM*	Not altered	*Off, Monitor Only, Electrocautery Mode*: constant tone *Monitor + Therapy*: beeping tone	*Inhibit Therapy:* detection remains active *Store EGM*: after 60 days or first EGM storage via magnet: automatic conversion to *Inhibit Therapy*
Medtronic	Detection and therapy inhibited	Not altered	Not programmable	Not altered	10 s continuous: normal function 30 s intermittent on–off (“truck backing up”): low-urgency alert 30 s alternating high–low frequency (“French police car”): high-urgency alert	None
Microport (former Sorin)	Detection and therapy inhibited	Altered	Not programmable	AV delay not altered, VV delay set to 0 ms	None	Pacing with 6V @ 1 ms for each chamber Paradym, Intensia: pacing at magnet rate; exiting magnet mode: 6 cycles at magnet rate with 95 ms AV delay and programmed output → 2 asynchronous cycles as permanently programmed → permanent programming Sensor (R-function) is disabled Therapy Inhibition may extend up to 2.5 min after magnet removal if a charge occurred just before magnet application Device in Mode switch: pacing at permanently programmed mode independently of underlying rhythm

* Avant, Gallant, Entrant, Neutrino. ^n^ Nominal programming. AV—atrioventricular; BOL—Beginning Of Life; EGM—intracardiac electrogram; EOL—End Of Life; h—hour(s); s—second(s); SJM—St. Jude Medical.

**Table 5:** Electrical Reset in CIED.

Manufacturer	Pacing Mode Brady/ Tachy	Pacing Rate Brady/Tachy	Pacing Polarity Brady/Tachy	Pacing Output Brady/Tachy	Remarks
Abbott (former SJM)	VVI/VVI	67 ppm/67 ppm	unipolar/ bipolar	5 V @ 0.6 ms/ 5 V @ 0.6 ms	CRT-D: LV pacing from tip to RV Ring (anodal capture possible) CRT-P: unipolar LV pacing Victory, Zephyr, Identity, Verity PM: pacing at 67.5 ppm, 4 V @ 0.6 ms
Biotronik	VVI/VVI	70 ppm/70 ppm	unipolar/ bipolar	7.5 V @ 1.5 ms/ 5 V @ 0.5 ms	In CRT-D: LV-output 4.8 V @ 0.5 ms
Boston Scientific	VVI/VVI	72.5 ppm/ 72.5 ppm	unipolar/ unipolar	5 V @ 1.0 ms/ 5 V @ 1.0 ms	In CRT: LV offset 0 ms, unipolar LV pacing
Medtronic	VVI/VVI	65 ppm/65 ppm	uniolar/ bipolar	6 V @ 1.5 ms/ 6 V @ 1.5 ms	Older models (Adapta/Versa/Sensia/Relia): bipolar pacing with 5 V @ 0.4 ms
Microport (former Sorin)	VVI/VVI	70 ppm/60 ppm	unipolar/ bipolar	5 V @ 0.5 ms/ 5 V @ 0.35 ms	-

Brady—pacemaker or CRT-P, SJM – St. Jude Medical, tachy—ICD or CRT-D.
